# What will it take to get to the heart of stigma in the context of HIV?

**DOI:** 10.1002/jia2.25934

**Published:** 2022-07-12

**Authors:** Lucy Stackpool‐Moore, Carmen H. Logie, Allanise Cloete, Finn Reygan

**Affiliations:** ^1^ IAS – International AIDS Society Geneva Switzerland; ^2^ Watipa Sydney New South Wales Australia; ^3^ Factor‐Inwentash Faculty of Social Work University of Toronto Toronto Ontario Canada; ^4^ Women's College Research Institute Women's College Hospital Toronto Ontario Canada; ^5^ Centre for Gender and Sexual Health Equity Vancouver British Columbia Canada; ^6^ Human and Social Capabilities Division Human Sciences Research Council Cape Town South Africa; ^7^ Human and Social Capabilities Division Human Sciences Research Council Pretoria South Africa

**Keywords:** community leadership, HIV, intersectionality, measurement, methods, Stigma

More than 40 years into the global HIV pandemic, we are still grappling with HIV‐related stigma and its intersections with other marginalized identities, health conditions and social practices. HIV‐related stigma, conceptualized as the devaluing, mistreatment and constrained access to power and opportunities experienced by people living with and associated with HIV, remains a critical concern inhibiting the HIV response [1]. Indeed, the UNAIDS Global AIDS Strategy explicitly describes the goal that “people living with HIV, key populations and people at risk of HIV enjoy human rights, equality and dignity, free of stigma and discrimination” to realize optimal HIV outcomes [2]. The inclusion of commitments towards eliminating HIV‐related stigma and discrimination within the Political Declaration agreed at the 2021 United Nations High‐Level Meeting on HIV/AIDS for the first time also signals a conducive global political environment for action at scale [3]. The time is now to renew and innovate responses to HIV‐related stigma, including taking the steps needed to ensure an enabling global policy environment.

Reducing stigma and alleviating its harmful effects is an essential ingredient of any effective national HIV response. Approaches can be informed by a focus on human rights, agency and intersectionality, which may be understood as a “discourse about identity that acknowledges how identities are constructed through the intersection of multiple dimensions” [4] and captures the complexities of social identities and social power. Contextual differences can be significant, and as evident from the work of the Global Partnership for Action to Eliminate all forms of HIV‐Related Stigma and Discrimination, it can be useful to focus on understanding how and where stigma manifests itself in specific settings for diverse communities in different geographies [5]. To succeed in reducing or alleviating its harmful effects, efforts must remain situated firmly within increased human rights realization for people living with and most affected by HIV. Research that focuses on stigma processes and their harmful impacts can also attend to the ways in which people exert individual and collective agency to resist and dismantle stigma, and form solidarity. A dual focus on stigma's harms *and* the ways in which people and communities navigate stigma can avoid perpetrating binary or simplistic notions of powerlessness, vulnerability and passivity, and instead calls attention to the nuances and fluidity of power dynamics [6]. A focus on “whole” selves can be informed by intersectionality theory to take into account interlocking systems of oppression—including stigma and discrimination [7, 8]. More could be learned from other sectors regarding how to understand and address HIV‐related stigma, including social ecologies of resilience [9, 10], activism [11] and civic engagement [12], community mobilization [13], collective impact [14], peer support and solidarity among persons living with HIV [15], and collective and self‐efficacy [16].

This Supplement on Getting to the heart of stigma across the HIV continuum of care aims to draw attention to HIV‐related and intersecting stigma and discrimination across the HIV prevention and care continuum. The articles contribute to consolidating the evidence base and provide a state‐of‐the field update about the latest concepts, innovative research methods and strategies to reduce stigma and/or ameliorate its harmful effects. Articles cover a variety of lived experiences of stigma; and at times, include examples of resilience, good practice and community leadership. Language is important, and the authors whose work is published in this Supplement have been encouraged to follow the latest terminology guidance from UNAIDS and to adopt person‐centred language, such as avoiding acronyms and using language that puts the person first (see, e.g., the People First Charter). The language used in research may in fact result in practice changes to engage person‐centred language in social and healthcare encounters [17]. Several papers in the Supplement include important methodological insights about the co‐creation of research and co‐production of knowledge, including with marginalized groups (see Brown et al. [18], Gamarel et al. [19], Tun et al. [20] and Collier et al. [21]). A partnership model between researchers and marginalized groups in the co‐creation of knowledge is increasingly influencing stigma research and is reflected in some of the studies in this Supplement. Such approaches foster knowledge production for greater impact and social change that are led by community researchers and/or more grounded in lived experiences. It is our hope that this Supplement informs efforts to address stigma and discrimination and ultimately improving quality of life and access to healthcare for people living with and most affected by HIV.

Studies in this issue examine the impact of HIV‐related stigma on the HIV prevention cascade. For instance, Hargreaves et al. [22] explore the association between stigma and HIV incidence through a nestled study within the PopART trials in Zambia and South Africa. They found no evidence of an association between HIV stigma and HIV incidence in the trials, suggesting that efforts to reduce new HIV infections and improve HIV prevention and treatment programmes may fail if HIV stigma is considered in isolation and are not complemented by a more holistic approach. In another paper, Atkins et al. [23] evaluated the factor structure of a pre‐exposure prophylaxis (PrEP)‐related stigma scale as part of a larger prospective cohort study nested within Kenya's Jilinde programme. They identified four dimensions of PrEP‐related stigma; and the scale demonstrated strong internal consistency, was positively correlated with depressive symptoms and negatively correlated with uptake of HIV services. Prevention cascade stigma research and practice should consider PrEP stigma alongside other prevention barriers.

Other papers focus on HIV‐related stigma impacts among people living with HIV. Johnson‐Peretz et al. [24] focus on schools in rural Africa as potential sites of stigma for young people. Authors apply a life‐course framework to explore a time of critical life stage transition, finding the young people in the study were already engaged in finding ways to manage their own healthcare, while refusing to internalize stigma, and were becoming invested with greater responsibility for their own, and their families’ health. Collier et al. [21] explore multi‐dimensional experiences of stigma among people living with HIV and Kaposi's sarcoma in Kenya. The intersection of HIV‐related, cancer‐related and skin disease‐related stigma was better understood using mixed‐methods approaches with people living with both HIV and cancer. Other studies explore stigma within broader structural determinants of health, such as poverty. For instance, Logie et al. [25] examined both food and housing insecurity as drivers of HIV‐related stigma, and present findings from a longitudinal engagement with a cohort of women living with HIV in Canada, finding resource scarcities linked with increased experiences of HIV‐related stigma.

Several papers in this Supplement focus on opportunities to address or reduce stigma among or for diverse groups of people. The paper by Pollack et al. [26] looks at work to reduce HIV‐related stigma and discrimination in healthcare settings in Vietnam, and their findings demonstrate the effectiveness of a multi‐pronged facility‐level intervention. Nyblade et al. [27] suggest that in order to get to the “heart of stigma,” efforts must understand and respond to both HIV and other intersecting stigma targeting sexual and gender diversity, and take a non‐siloed approach to training healthcare providers. Connecting within a focus on intersectionality, structural processes of stigma and practical opportunities to address biases within the healthcare system, their paper discusses findings and curriculum adaptation for a total health facility approach for stigma reduction. From a community perspective, Tun et al. [20] focus on transgender men and women in Nigeria and discuss how provider awareness of, and respect for individual gender identity is critical for optimal delivery of HIV and other health services for Nigerian transgender men and women.

Peer‐support and community leadership in challenging and researching stigma is essential to contributing to the robust evidence base of what works to respond to stigma. Makoni et al. [28] provide examples of the importance of community‐led monitoring in promoting accountability and better policy responses that meet the needs of the spectrum of diverse people living with and affected by HIV in Zimbabwe. Gamarel et al.’s [19] commentary proposes a status‐neutral approach for research with trans communities in the United States. The authors argue that although interventions focused on PrEP or antiretroviral therapy uptake and adherence have and will continue to benefit communities, these HIV “status‐segregated” interventions can perpetuate HIV stigma and other forms of oppression among those in most need of HIV programmes. They argue that segregating people into HIV prevention and HIV treatment research disrupts the organic and close kinship structures, and conclude by calling on funders to develop mechanisms that support the development and testing of HIV status‐neutral interventions. Brown et al.’s [18] community‐led innovation with systems thinking considers how to get to the heart of addressing stigma at scale. The authors present findings from a study adopting a systems perspective to understand how to tackle structural stigma via the Meaningful Involvement of People with HIV, while highlighting the challenges in demonstrating peer leadership from people living with HIV.

The Supplement also includes papers that review conceptual frameworks and measures used to evaluate stigma, including recommendations for different scales and approaches to robustly measure stigma and track change over time. Ferguson et al. [29] present findings from a global systematic review that highlight the gaps and diversity within existing measures and conceptual frameworks to address stigma. Finally, Golub and Fiskin's [30] commentary suggests that HIV researchers and practitioners have failed to fully specify or examine the mechanisms through which HIV service implementation itself may reinforce stigma and inequity.

Taken together, the articles in this Supplement offer insight into a range of health conditions, social identities, social determinants of health and life stages that shape lived experiences of stigma. It also provides insight into wide‐ranging methodologies, including qualitative, quantitative, systems mapping and systematic reviews, that were employed to generate new insights into the complexity of stigma. Getting to the heart of stigma requires engagement across methods, conceptual frameworks and impacted communities to understand what factors are most important to translate research to action to advance human rights and equity.

## COMPETING INTERESTS

The authors declare no competing interests.

## AUTHORS’ CONTRIBUTIONS

The Editorial was conceptualized by LSM, CL and AC. LSM and CL wrote the first draft of the Editorial. AC and FR reviewed and contributed additional material. All authors reviewed and revised the Editorial before final submission.

## FUNDING

This work was supported from the Bill and Melinda Gates Foundation, Investment number INV‐004364.
